# Tibia Nailing Using Modular Stand: A Technical Note

**DOI:** 10.7759/cureus.24801

**Published:** 2022-05-07

**Authors:** Rajendra Chandak, Aditya L Kekatpure, Rahul Agrawal, Aashay Kekatpure

**Affiliations:** 1 Orthopaedic Surgery, Chandak Nursing Home, Nagpur, IND; 2 Orthopaedic Surgery, Jawaharlal Nehru Medical College, Datta Meghe Institute of Medical Sciences (DMIMS), Wardha, IND; 3 Orthopaedic Surgery, NKP Salve Institute of Medical Sciences and Research Centre and Lata Mangeshkar Hospital, Nagpur, IND

**Keywords:** intraoperative fluoroscopy, ergonomic design, tibia shaft fracture, modular tibial stand, tibia nailing

## Abstract

Interlocking nailing is a well-established procedure for managing unstable tibial shaft fractures. Closed reduction and internal fixation of the tibial shaft fractures require ease of intraoperative positioning, maneuvering, and biplane imaging. We describe the use of an innovative modular tibia-nailing stand, which greatly enhances the ergonomics of the tibia nailing procedure.

## Introduction

Closed tibia nailing is a popular, established, and commonly performed operative procedure for the treatment of tibial shaft fractures [1]. For improved outcomes, optimum fracture alignment and rotational correction play an important role [2]. Intraoperative fracture alignment is usually obtained by applying traction along the long axis of the leg manually or by distractor assistance. Final reduction and nail position depend on the accuracy of guidewire placement, which requires biplane imaging [3]. To achieve this, the procedure is performed on a radiolucent operation table with traction being applied either manually or on traction assembly [4,5]. Even though these techniques are well established and commonly used, there are few associated difficulties in patient positioning, maneuvering, and intraoperative biplane imaging. The availability of a fracture table exclusive to the tibia is also an issue. It is not portable and requires significantly longer positioning time than manual traction, making it inconvenient and time-consuming [6]. Also, it does not allow sequential or simultaneous procedures in a polytrauma patient with multiple lower limb injuries without re-positioning or re-draping. Manual traction overcomes all these drawbacks of standard fracture table traction and significantly decreases the operative time [3]. Manual traction can be applied using a radiolucent foam triangle, adjustable knee and tibial positioner device, tibial and femur triangles, or hanging free leg technique [5]. However, the inventory required for the manual traction using the above-mentioned commercially available devices is cumbersome, and maintaining operative field sterility can also be an issue.

We have developed a simple autoclavable modular tibial stand to overcome these technical difficulties with manual traction application in the tibia nailing procedure.

## Technical report

**Figure 1:**
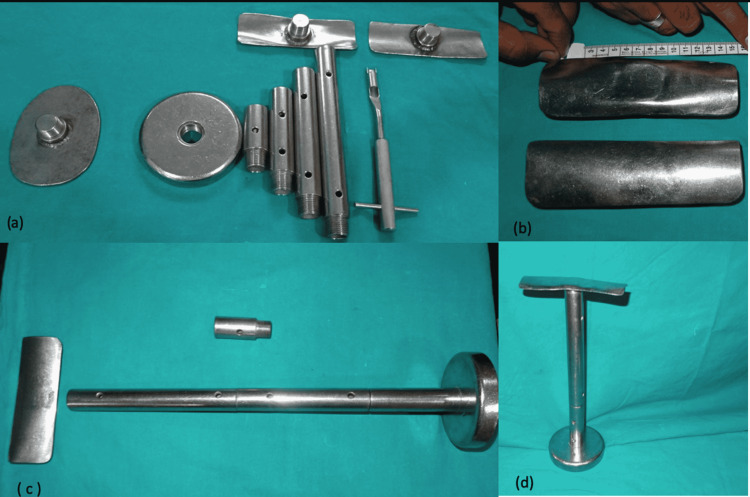
Components of modular tibia stand. (a) Modular tibial stand with the four detachable cylindrical attachments of various heights, tibial baseplate, and top resting plate; (b) Knee resting plates; (c) Maximum tibial stand length; (d) Complete modular tibial stand.

**Figure 2:**
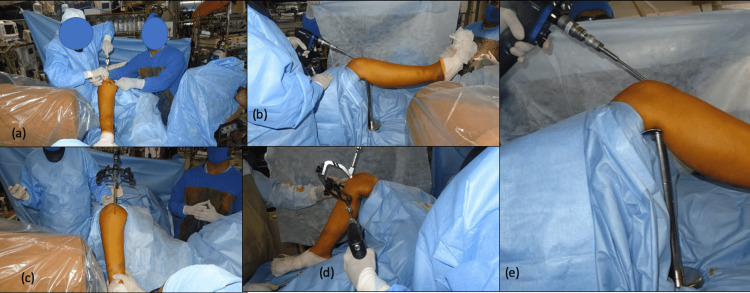
Various steps of tibia nailing with modular tibial stand. (a) Guidewire insertion under fluoroscopy control; (b) Reaming in extension (needed in proximal tibia fractures); (c) Nail insertion with the jig for proximal locking; (d) Proximal tibia locking (e) Reaming in flexion.

Patients are operated on under suitable anesthesia (neuraxial block or general anesthesia as required) in the supine position, under tourniquet control. After sterile draping and prepping, ensured a free position of drapes such that the knee can be flexed up to 90 degrees. Next, the foot is draped separately. A modular tibial stand is assembled (Figure 1) and used throughout the procedure to keep the knee in various degrees of flexion based on the requirement (Figure 2).

**Figure 3:**
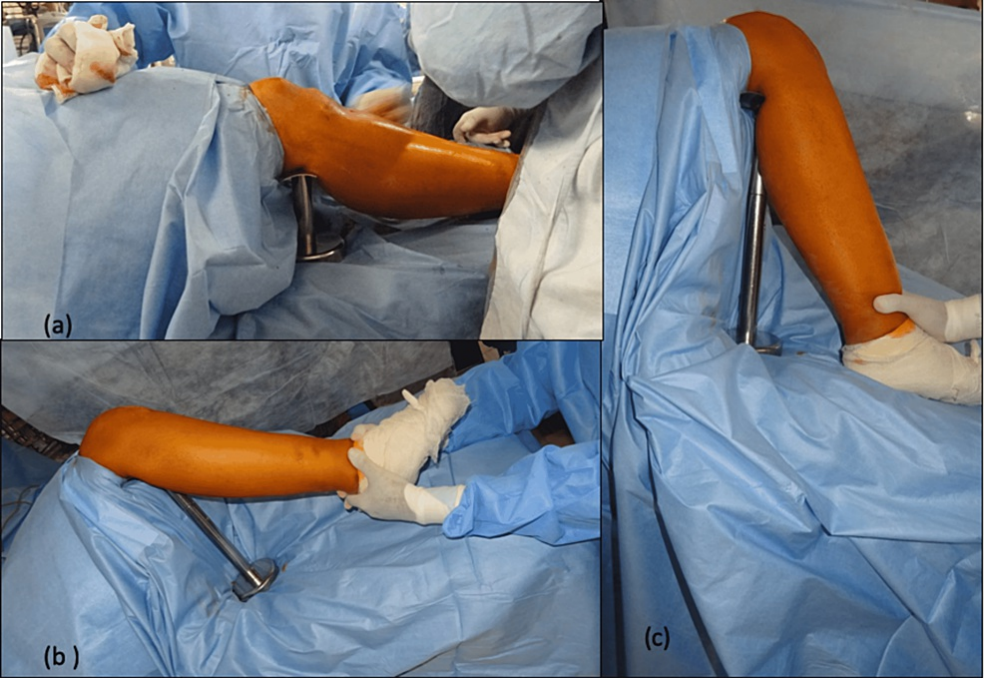
Various degrees of knee flexion possible with modular tibia stand. (a) Thirty degrees of knee flexion with the smallest attachment of tibial stand is required during entry point incision and during closure to maintain adequate tissue tension; (b) Modular tibial stand for applying on-table traction to align fracture; (c) 100 to 110 degrees of knee flexion possible with the modular tibial stand, required during guidewire and nail insertion.

Routine steps for tibia nailing are done. Various degrees of knee flexion possible with a modular tibial stand is described pictographically (Figure 3).

**Figure 4:**
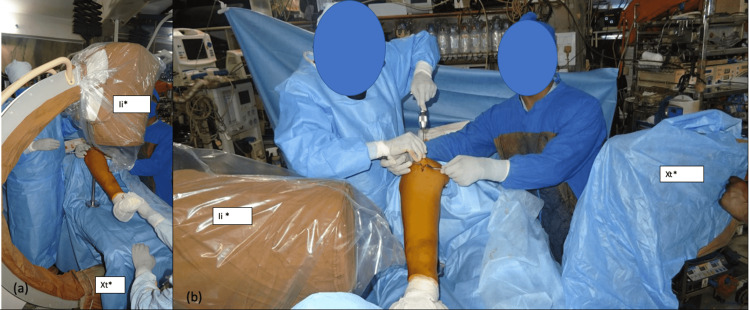
Ease of intraoperative biplane imaging with modular tibial stand. (a) Intraoperative anteroposterior imaging; (b) Intraoperative lateral imaging. *Xt: X-ray Tube *Ii: Image Intensifier

Intraoperative biplane imaging is done throughout the procedure, as required (Figure 4).

**Figure 5:**
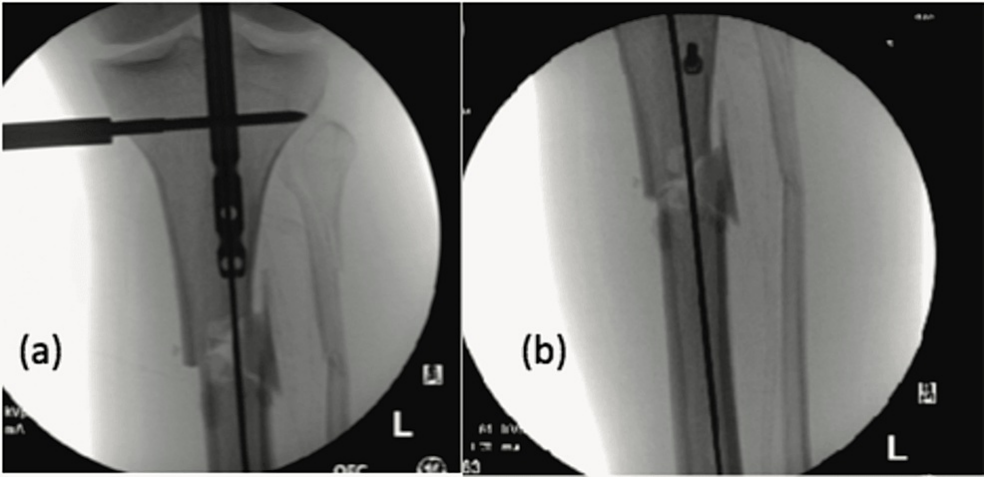
Intraoperative fluoroscopic images with modular tibial stand. (a) anterior-posterior (AP) view; (b) lateral view.

The important peculiarities of the tibial stand and its advantages have been described in Table 1. Even though it is metallic, it does not hinder intraoperative fluoroscopic views, as shown in Figure 5.

**Table 1:** Peculiar features and advantages of the tibial stand.

Sr No.	Features	Advantages
1.	Made of stainless steel	Autoclavable
2.	Heavy tibial base plate	Improves stability traction counter traction ability; Good rotation and varus/valgus control; The scrubbed surgical assistant stabilizes the cylindrical block with one hand, thereby preventing intraoperative toggling of the leg when giving traction.
3.	Adjustable, slotted four cylindrical blocks of various lengths	Provides modularity in the height of the tibial stand as required during the procedure; During skin incision and closure: 30-degree flexion is desired, which can be obtained using the smallest size cylinder; During guidewire negotiation, traction-countertraction application for fracture manipulation, and nail insertion: 90 to 110 degrees knee flexion is desired and can be obtained by using all the four slotted cylinders; Nailing in extension is easily possible.
4.	Wide and narrow top plate	The top plate is convex, smooth, and wrapped with a thick gauze to avoid damage to popliteal structures during traction application; Does not hinder the intraoperative fluoroscopy view of the proximal nail insertion site (Figure 5).

## Discussion

Closed tibia nailing is a popular and commonly performed operative procedure for treating tibial fractures [1]. For better postoperative outcomes, optimum fracture alignment, ease of intraoperative biplane imaging, and rotational control play an important role [2]. Intraoperative fracture alignment is obtained by applying traction in the long axis of the leg during guidewire and interlock nail negotiation [3]. There are various intraoperative positioning and traction application techniques during the tibia nailing [4,5]. These techniques have been summarized with the pro and cons of each technique (Table 2).

**Table 2:** Various methods of intraoperative positioning and traction application during intramedullary nailing of the tibia.

	Traction through a smooth calcaneal pin	Pinless calcaneal traction by foot strapping	Adjustable knee and tibial positioner	Readymade tibial triangles	Hanging free leg technique	Fine wire frame assisted IM nailing	Modular tibial stand
Method of traction	Traction table	Traction table	Manual	Manual	Gravity assisted and manual	Distraction over circular frame	Manual
Traction table required	Yes	Yes	No	No	No	No	No
Patient positioning time	Increased	Increased	Less	Less			
Autoclavable	Yes	No	Yes	Yes	Not Applicable	Yes	Yes
Multiple simultaneous or sequential procedures in a polytrauma patient without redraping	Not possible	Not possible	Possible	Possible	Possible	Possible	Easy
Access to contralateral lower limb for comparison of length and rotation	Difficult	Difficult	Easy and rapid	Easy and rapid	Difficult	Easy	Easy and rapid
Associated complications/Limitations	Subtalar joint encroachment; Over distraction; Calcaneal pin site discharge	Skin excoriation in case the skin is friable or paper-thin; Difficult to apply if there is associated lower extremity injury like Bimalleolar fracture	Difficulty in sterilization	Cost; Need to maintain complete range of inventory	Exaggerated lower extremity edema; Risk of compartment syndrome and common peroneal nerve palsy in the contralateral leg in lithotomy position	Additional procedure of half-frame attachment; Risk of neurovascular injury during insertion of fine wires in the proximal and distal segments; Difficulty in intraoperative biplane imaging	Nil noticed in our series

It has been shown that manual traction can significantly reduce the operating time compared to fracture table traction without compromising fracture alignment and outcome [3]. However, the commercially available inventory required for applying intraoperative traction can be cumbersome and on-table space-occupying [6].

We have found that modular tibial stand overcomes the difficulties associated with manual traction with excellent on-table biplane imaging and good axial and rotational control of the fracture possible by the assistant applying longitudinal traction. The assistant applying manual traction can easily control the intraoperative fracture site's rotational and axial alignment. The heavy tibial base plate provides improved stability, traction/countertraction ability, and good varus/valgus control. The height of the stand can be easily altered as per requirement because of the modularity of the various size cylindrical blocks of various lengths. Being made of stainless steel, it can be autoclaved easily.

Over the last 10 years, we have operated on over 600 cases of all tibial interlocking nailing using a modular tibial stand at our center. With the use of a tibial stand, we have found that the ergonomic of tibia nailing procedure has significantly improved in terms of reduced operative time, ease of intraoperative fluoroscopy, and traction application.

## Conclusions

Obtaining satisfactory fluoroscopic images during the tibia nailing procedure and effective fracture site manipulation throughout the treatment necessitates proper intraoperative limb positioning. The use of a modular tibial stand, in our experience, takes care of both these concerns and assures that the results are repeatable. This could be a useful and safe alternative to the currently available limb positioning modalities during tibia-closed intramedullary nailing.
